# Navigating sleep apnea in Scandinavia - a journey of contrasts

**DOI:** 10.1080/17482631.2026.2629877

**Published:** 2026-02-11

**Authors:** Laila Steen, Heidi Jerpseth, Nils Henrik Holmedahl, Kate Sellen, Kristin Halvorsen

**Affiliations:** a Department of Product Design, Faculty of Art and Design, Oslo Metropolitan University, Oslo, Norway; b Department of Nursing and Health Promotion, Faculty of Health Sciences, Oslo Metropolitan University, Oslo, Norway; c Institute of Aviation Medicine, Oslo, Norway; d Department of Systems Design, University of Waterloo, Waterloo, Ontario, Canada; e Faculty of Design, OCAD University, Toronto, Ontario, Canada

**Keywords:** Obstructive sleep apnea (OSA), patient experience, Co-Design, reflexive thematic analysis, CPAP therapy, healthcare services, qualitative research, service improvement

## Abstract

**Study objectives:**

Obstructive sleep apnea is a prevalent condition with substantial physical, psychological, and social burdens, yet limited research explores how patients experience sleep apnea–related services in the Scandinavian context. This study aims to examine lived experiences of diagnosis, treatment, and follow-up services in Southeast Norway to identify opportunities for service improvement.

**Methods:**

Using a Co‑Design approach, the study examined assessment and treatment services through participatory workshops and semi‑structured interviews with ten (10) participants. Reflexive thematic analysis, guided by Braun and Clarke’s framework, supported an inductive development of themes, refined through participant feedback and comparison across data sources.

**Results:**

Four themes emerged: (1) The CPAP machine - a lifeline and a toll, (2) Forsaken but not fallen - carrying the weight of one’s own care, (3) Healthcare providers - obstacle and ally, and (4) Life on hold - the emotional and social impact. Differences in workshop and interview participants suggest more personalised services may influence experiences and outcomes.

**Conclusions:**

Our analysis highlights the need for more tailored, responsive and coordinated services that reflect to the diverse physical and psychosocial realities of people living with obstructive sleep apnea. These exploratory findings will inform subsequent Co-Design work to improve service models.

## Statement of significance

Obstructive sleep apnea is a widespread disorder that poses significant physical, psychological, and social challenges. This study provides novel insights into peoples lived experiences with diagnosis, treatment, and follow-up services in a Scandinavian context, an area with limited prior research. Using Co-Design methodology, it highlights challenges in current care models, particularly the burdens of CPAP treatment, the personal responsibility patients bear, and the complex roles of healthcare providers. Findings underscore the need for more personalized, holistic service models that address not only clinical but also psychosocial aspects of obstructive sleep apnea care. This work offers valuable guidance for patient-centered approaches and health service improvement for this population.

## Introduction

Obstructive sleep apnoea (OSA) is a common disorder estimated to affect almost 1 billion people globally (Benjafield et al., 2019). The disorder is characterised by episodes of partial or complete obstruction of the upper airways during sleep, causing arousal of the brain and sympathetic activation in response to oxygen desaturation in the blood. Repeated episodes of apnoeas may result in fragmented sleep and overall lack of restorative sleep. People may experience symptoms such as severe drowsiness, headaches, and loud snoring, while some are asymptomatic (Park et al., 2011). There is also growing evidence that OSA is associated with adverse long-term cardiovascular effects and metabolic dysfunction (Hirani & Smiley, 2023). Early diagnosis and treatment of sleep apnoea may therefore help reduce associated morbidity and mortality, in addition to increasing quality of life (Holt et al., 2018).

The current most efficacious and commonly prescribed treatment for OSA is continuous positive airway pressure (CPAP) therapy, in combination with services to support the fitting of CPAP machines, and training to support people in home-based CPAP therapy (Hirani & Smiley, 2023; Jacobsen et al., 2017; Platon et al., 2023). Despite being the gold-standard treatment, long-term adherence with CPAP is reported as low as 25.7% in people with mild OSA (Qiao et al., 2023). The low adherence is attributed to various factors, such as lack of experienced effectiveness, inconvenience of use, as well as physical and psychological discomfort affecting use (Brown et al., 2021; Engleman et al., 1999).

CPAP therapy exemplifies technology-enabled home-based care, an area of focus as the healthcare industry grapples with increasing demands driven by a growing elderly population with chronic care needs. While home-based care technologies offer benefits such as patient empowerment, independence, and a sense of safety through active symptom management and monitoring, they also present challenges, particularly regarding technical and practical usability (Leonardsen et al., 2020). These technologies have been widely implemented for managing chronic conditions like diabetes and Chronic Obstructive Pulmonary Disease. As experience with these systems has grown, research on patient experiences highlights the importance of involving patients in the development and planning of these solutions as a key component of quality improvement efforts.

Sleep apnoea has been shown to significantly affect individuals’ psychological well-being and overall functioning. A systematic synthesis of qualitative research on the lived experience of people with sleep apnoea found that the condition results in a wide range of daytime physical and mental symptoms, which can severely impair both physical and psychosocial functioning (Chua et al., 2023) These symptoms, such as daytime drowsiness, stress, and anxiety, are closely intertwined and influence not only daily functioning but also patients’ perceptions of their health and ability to manage the condition. Given the heterogeneous nature of sleep apnoea in both symptom presentation and treatment outcomes, a more biopsychosocial approach that emphasises holistic, person-centred assessment and care has been recommended (Crawford et al., 2014; Engleman & Wild, 2003; Hilbert & Yaggi, 2018).

Despite high prevalence, little research has been done on people with OSA’s experience with sleep apnoea and current service offerings in the Scandinavian context (Chua et al., 2023).

The aim of this study was to explore the lived experiences of people with OSA in relation to diagnosis, treatment, and follow-up services in the Southeast region of Norway. The reported findings are intended to form the empirical basis for later workshops where people with OSA and health care staff will define and ideate suggestions for service improvements together.

## Methods

### Study design

This article presents findings from Sub-Study 2 of a PhD project that uses Co-Design to explore diverse perspectives on sleep apnoea and inform future service recommendations. The context for the study was services for people undergoing assessment and treatment for sleep apnoea in Southeast Norway. The project follows a Co-Design methodology with an exploratory approach aimed at identifying service-related challenges and opportunities (Pfannstiel & Christoph, 2019; Sanders & Stappers, 2008). Applying Co-Design techniques within a service design framework, the project consists of several phases to engage stakeholders, gather experiences, identify needs and challenges, and develop service improvements with the aim of implementing them in practice (Burkett, 2016; Council, 2025). In this context, service design is understood as a human-centred, holistic, creative, and iterative approach to shaping future services (Patrício et al., 2017; Schneider et al., 2010).

A core principle of Co-Design is the active involvement of end-users through participatory activities to ensure that solutions are aligned with their needs. Co-Design emphasises close collaboration between stakeholders, such as service users and providers, throughout the design process (Sanders & Stappers, 2008), and is widely recognised for achieving meaningful impact in health research (Greenhalgh et al., 2016; Peters et al., 2024). Co-Design emphasises the value of “doing” and “making” through tools like storyboards, diaries, and prototypes as alternative forms of expression that can help unlock tacit knowledge and deeper insights among participants; ideas and experiences that go beyond what they might be able to articulate in words (Moll et al., 2020; Morrison & Dearden, 2013; Sanders & Stappers, 2014). In this project, we used journey maps, an increasingly common tool in health services research to highlight factors influencing the care experience and to identify gaps and opportunities for service improvement (Davies et al., 2023). The patient journey-map, depicting the steps, encounters, activities and touchpoints with the service from the patients' point of view, was used as a tool in the individual interviews to help trigger recall and discussions (Davies et al., 2023; Schneider et al., 2010).

**Figure 1.**
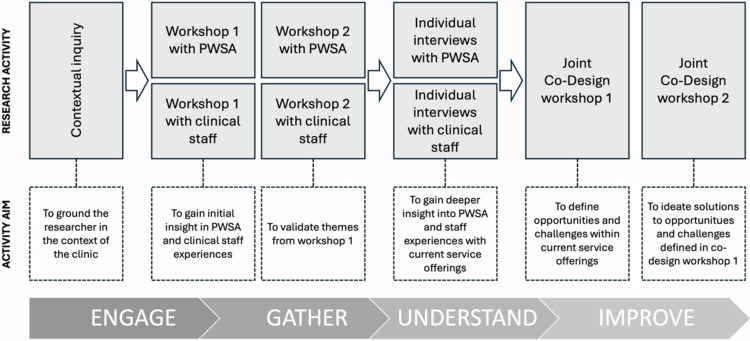
The flow chart illustrates the specific research activities, the aim of each activity, how stakeholders, including people with sleep apnoea (PWSA) and clinical staff, are involved, and the Co-Design principles underpinning each stage.

A flow chart of the project is presented in Figure 1. The flow chart illustrates the specific research activities, the aim of each activity, how stakeholders are involved, and the overarching Co-Design principles underpinning each stage. While the figure presents a linear process, it should be understood as responsive and iterative in practice. This article presents the insight from workshops (group sessions 1 and 2) and individual interviews with people with sleep apnoea.

### Context

In Norway, the assessment and treatment of sleep apnoea is subject to the secondary healthcare services based on referral from a persons’ primary care doctor. In most of the larger secondary care hospitals across the country the wait times for initial assessment and diagnosis of OSA are long, with 22 out of 27 institutions reporting wait times of 12 weeks or more (Helsenorge, 2025). The regional health authorities increase capacity to meet demand and reduce wait times through tender agreements with private healthcare providers.

**Figure 2.**
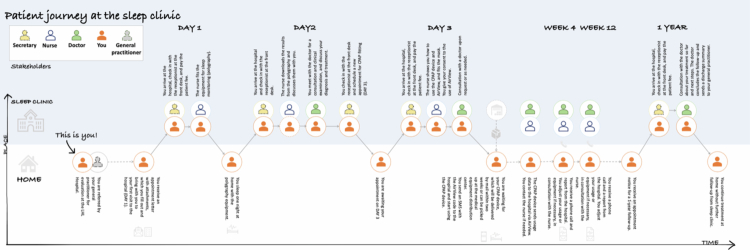
Patient journey map through assessment and follow-up at the sleep clinic.

This study was conducted with an outpatient clinic within a private, non-profit hospital in Southeast Norway as a vantage point. The clinic provides assessment and treatment for sleep apnoea under a tender agreement, meaning patients are referred as part of the public healthcare system. During this project, the wait times at the clinic was 5 weeks. The sleep clinic provides three initial appointments: a preliminary assessment, a consultation to review home-based polygraphy results for diagnostic purposes, and a CPAP fitting. This is followed by remote monitoring via AirView™, with progress reports issued to patients at weeks 4 and 12. AirView™ is a cloud-based platform used to remotely monitor and manage data from sleep and respiratory therapy devices (ResMed, 2025). The service concludes with an in-person follow-up appointment at the clinic one year after treatment initiation (see Figure 2).

As the clinical service at the hospital had only been operational for a short time, it was necessary to also look beyond the sleep clinic to gain broader insights from people with OSA who had been living with the diagnosis and engaged with health services for a longer period, and who had completed treatment and follow-up programs at other institutions in Southeast Norway.

### Recruitment

Participants for the workshops were recruited through convenience sampling. Three participants were recruited through the The Norwegian Sleep Association, which is a patient organisation that advocates for individuals with sleep disorders. An information letter was forwarded to all members of the association with a request to contact the first author (LS) directly to express interest in participating. Two participants were identified in conjunction with the sleep clinic staff and contacted by the first author with a request to participate. These were both people who had been diagnosed for some time and had experience with service offerings elsewhere. One additional participant was recruited at a research dissemination event after expressing interest. The lead researcher then followed up with all participants to provide further information, complete consent procedures, and to schedule time for the interview.

Participants were recruited from the sleep clinic for the individual interviews. They received an information letter from the clinical staff at the end of the 1-year follow-up appointment, asking them to contact the first author on a voluntary basis via email or phone to express interest in participating. The first author followed up with further information and consent procedures, and to schedule time for the interview via email.

Given the limited geographical and demographic scope of the study, detailed participant data, including diagnostic specifics, apnoea–hypopnea index (AHI) scores, and compliance metrics, are not reported to preserve anonymity. All participants, however, represented individuals with clinically significant sleep apnoea, evidenced by AHI scores of ≥5 and self-reported daytime symptoms sufficient to qualify them for diagnosis and treatment (Bathgate & Edinger, 2019).

### Data collection

The PhD project began with a contextual enquiry phase, followed by workshops and individual interviews with people with OSA. The goal of the contextual enquiry was to “ground” the researcher in the life and context of the clinic, their patients and staff (Holtzblatt & Beyer, 2017). In this project, the contextual enquiry served the particular purpose of familiarising first author, whose background is primarily in design, with the clinical context, patient population and diagnosis. The contextual enquiry gave the first author an overview of the service at thesleep clinic, and a preliminary understanding of opportunities and challenges faced by people who are diagnosed with sleep apnoea. The contextual enquiry led to the creation of a patient journey-map (Figure 2) that was used in the individual interviews.

#### Workshops

Workshops were organised to initially engage with people already diagnosed with sleep apnoea, who had received assessment, treatment and follow-up services at other institutions in the Southeast region of Norway. The group format was chosen in the hopes that the social interaction among the participants would help drive the conversation and contribute to more spontaneous expressions and discussions (Brinkmann, 2013). A second workshop was conducted to validate insights and elaborate on central themes based on a preliminary analysis of the findings from the first round. Initially, six (6) people agreed to participate, but one failed to show, so five (5) people were interviewed (1 woman and 4 men). Three (3) people (all men) participated in the second, in-person group interview. One participant failed to show here, while one was unable to attend in-person and contributed via email. Two members from the research team facilitated both workshops, with one serving as moderator (LS) and the other as rapporteur (KH). The participants were asked to introduce themselves and tell their own “sleep apnoea story”. The participants were also provided with paper and writing tools if they wanted to illustrate concepts. The workshop lasted 3 hours. In the second workshop, the participants were presented with a thematic summary of the findings from the first interview. The findings were based on an initial thematic analysis, using Braun and Clarks framework for reflexive thematic analysis (2019). Both workshops were audio recorded and later transcribed.

#### Individual interviews

**Figure 3.**
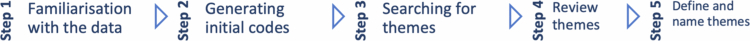
Step 1-5 in Braun & Clarks framework for thematic analysis.

Five (5) participants, (1 woman and 4 men), took part in the individual interviews. Due to the ongoing Covid-19 pandemic, no in-person interviews could be conducted with patients, so the participants were given the option of interview by phone or by videoconference (Zoom). The interviews were semi-structured, with the participants being asked to “tell their sleep apnoea story”. After the interviewee had spoken freely for some time, the interviewer asked more specific questions related to topics that were unclear or central themes from the workshops that had not been raised. Towards the end of the interview, the interviewee was shown a patient journey-map. This was a modified version of the clinical journey map, where only the patient pathway was illustrated (ref Figure 3). This was to help trigger recall and discuss questions specific to the service at the sleep clinic and subsequent follow-up services. In one instance the interviewee was shown the journey map ahead of the interview as they could only participate via phone and thus had to receive the journey map via email.

### Data analysis

Braun and Clarks (2019a, 2019b) framework for reflexive thematic analysis was used to analyse the data from both the workshops and the individual interviews. The reflexive orientation of Braun and Clarke’s approach aligns well with the iterative and exploratory nature of co-design, as both emphasise ongoing interpretation, responsiveness, and the value of emerging insights (Braun & Clarke, 2019a; Braun & Clarke, 2024; Braun, 2024). Data from the two activities were analysed separately before they were compared and collated into the results. Braun and Clarks framework consists of six steps, from familiarisation with the data to producing a report. Steps 1-5 are illustrated in Figure 3. Details of each analytic phase are summarised in Table A1. As a first step in the analysis, the first author listened to audio recordings and read transcripts to become familiar with the data and note down initial observations. The notes reflected some of the key research questions, such as the experiences with current service offering, key concerns for patients, common issues or challenges they face, and what they wish could be better. The lead researcher then generated codes based on smaller sets of data that shared the same meaning, such as experiences with general practitioners (GPs). To ensure analytical rigour, the transcripts, coding and early interpretations were reviewed and discussed in regular meetings with members of the research team. The process was primarily inductive, meaning the codes were generated in a “bottom-up” approach from the data. This was done by keeping the analysis “superficial” and generating semantic codes using the language of the participants as labels. For instance, repeated words or phrases could be a code, such as “new life”, referring to how some patients reported to have a new life after treatment. However, the analysis became more deductive with each cycle as the data was reviewed with the research questions in mind through a service design lens, meaning the researcher was primarily looking for meaning related to patient experiences with the service that had the potential to be met or resolved through service improvement, rather than for instance strictly personal factors such as personal relationships. Based on these codes, candidate themes were formed. A preliminary analysis of the first workshop was presented to the patients in workshop 2. An important consideration in this part of the analysis was to not go “too far” by for instance looking for latent meaning but rather stay close to the language of the participants precisely so that they could be validated with the participants while still sounding familiar. Thus, some of the codes became themes themselves, while some codes collapsed into “new” themes. Each theme was presented to the participants with a description and quotes to illustrate the meanings during the second workshop. The themes were then revised based on the feedback from the participants during the second workshop. In the instance where new information emerged during this interview, it was added to the initial data set for further analysis. The return of preliminary findings to participants in workshop 2 exemplifies the emphasis on iterative, joint sense-making and validation of Co-Design methodology. Throughout this stage, multiple members of the research team also reviewed the coding structure and emerging themes to enhance reflexivity and strengthen the analysis. The analysis of the individual interviews followed the same steps as the workshop data; however, due to the pandemic we did not have the opportunity to review the preliminary findings with the participants. As a result, the analysis was carried out entirely by the research team. In the final stage, the initial themes generated from both groups were compared and integrated into a set of overarching themes. In line with Braun and Clarke’s framework for reflexive thematic analysis, (Braun & Clarke, 2019b; Braun et al., 2019; Braun, 2024) the analysis was considered complete once the researchers agreed that the themes were sufficiently developed and represented well-supported, coherent, and meaningful interpretations of the data. Discrepancies between workshop and interview data were interpreted as meaningful differences that informed the analysis and are presented in the results. Furthermore, consistent with Co-Design principles, these themes will not serve as final outputs but as generative insights. They will be further explored and developed in forthcoming workshops, where stakeholders will collaboratively translate them into tangible service recommendations, ensuring that any proposed improvements reflect both patient experiences and clinical feasibility.

### Researcher reflexivity

The primary author’s background in design shaped their interpretive lens, particularly through the intuitive use of collaborative processes and tangible tools, such as journey maps, to explore patient experiences. This perspective also guided attention during the analytical process to relational dynamics and touchpoints in service pathways. The wider team, comprising clinical and health science experts alongside a design researcher with extensive health Co-Design experience, brought complementary perspectives. We argue that the cross-disciplinary composition of the authors mirrors the interdisciplinary nature of the project, fostering reflexive dialogue, broadening the analytical lens, and strengthening the credibility of the interpretations.

### Research ethics

All the participants in the study were given oral and written information about the project and signed an informed consent form. The participants were informed about the option to withdraw from the study up until the data was analysed. The Regional Committees for Medical and Health Research Ethics (REC) exempted the project from ethics approval (reference number 2018/1096) since it is not considered medical or health-related research under Section 2 of the Norwegian Health Research Act. The data was stored in accordance with the applicable rules and guidelines for storing research data and the project was approved by the Norwegian Agency for Shared Services in Education and Research, approval number 60692 (Sikt, n.d.)

## Results

Based on our analysis we constructed four main themes. Table I provides a summary of the themes, while the following sections elaborate on each theme in detail, illustrating the findings with selected quotes to convey the depth and nuance of participants’ experiences.

**Table I.** Themes from workshops and individual interviews.

Theme	Description	
*The CPAP Machine—a Lifeline and a Toll*		A source of relief, dependency, vulnerability and frustration.
*“Forsaken but not fallen” - Carrying the Weight of One’s Own Care*		People navigate diagnosis and treatment “independently” but search for alternatives “alone”.
*Healthcare Providers—an Obstacle and an Ally*		Healthcare provider's, particularly GPs, play a crucial role in shaping participants ´ overall experience.
*Life on Hold—The Emotional and Social Impact*		Some reclaim quality of life, while some carry the weight of unmet expectations.

### The CPAP machine—a lifeline and a toll

Across workshops and interviews, CPAP emerged a central theme, seen as vital for symptom relief yet burdened by discomfort and practical challenges. Discussions revealed that for individuals living with sleep apnoea, the CPAP is not merely a medical device but an essential component of daily life, often associated with contrasting feelings of dependency, vulnerability, relief and frustration.

Several participants viewed the CPAP machine as a lifeline; a life-changing therapy that is seen as a crucial tool for managing sleep apnoea and something they depend on for daily functioning. Meanwhile, it is also described as a toll, as its use often comes with significant challenges, including discomfort, practical difficulties, and varying experienced effectiveness, with the latter seeming to shape the quality and nature of the relationship with the machine.

One participant in the workshop group described a conflicted and frustrating relationship with the CPAP machine, acknowledging its potential benefits, yet struggling to use it effectively. They spoke about forcing themselves to wear the device despite being unable to sleep with it, which in turn meant that the machine couldn’t accurately record any potential breathing pauses. This tension between effort and outcome is captured in their words:

*"It’s not certain that I’ve had any breathing pauses at all, because I’m lying awake. Forcing myself to keep it on."*

The conversations in both the workshops and the individual interviews reflected how maintaining, cleaning, and travelling with the CPAP machine adds to the daily workload for participants. Participants in both groups expressed concern for how breakdowns or failures can directly impact health, with replacements requiring patient initiative:

Workshop participants:

*"When the machine starts to fail, your condition may suffer."*

*"When the mask broke, I had to call [the hospital] and wait for them to send a replacement. It wasn’t quick, and in the meantime, I was back to poor sleep and feeling terrible during the day."*

One workshop participant voiced concern about the challenges of maintaining or replacing the device if their health were to decline, potentially leaving them reliant on home nurses or others who might lack the necessary experience.

Participants in the sleep clinic interviews seem to have overcome some of these challenges due to closer follow-up and one-to-one demonstrations:

*"At first, I struggled with the mask—it was uncomfortable, and I couldn’t sleep well. But the staff at [the sleep clinic] were so supportive. They adjusted the settings, helped me find a mask that fit better, and explained how to clean and maintain it. It made all the difference. Now, it feels natural, and I can’t imagine sleeping without it."*

One participant reflected on the potential emotional and psychological downsides of having constant access to the detailed information about their sleep quality that the CPAP machine offers:

*"Not everyone benefits from seeing this information, says the sleep association* [participant referring to the Norwegian Association of Sleep Disorders]*, and I fully understand that. Because everyone will experience something that isn’t good during the night. And some take it more seriously than others. If they see 10 breathing pauses, they might think, 'That was ten times I could have [died]."*

### “Forsaken but not fallen” - carrying the weight of one’s own care

The second theme highlights the tensions within participants' experiences and concerns about the extent of personal responsibility and effort required to obtain a diagnosis and manage treatment. Many described a sense of being abandoned by the healthcare system, contrasted with a strong personal resolve to take control of their care and find their own path forward.

Participants in both groups described carrying significant responsibility for their own care when it came to seeking a diagnosis. The experience diverged between the two groups when it came to managing follow-up and treatment adjustments. The participants in the sleep clinic interviews felt more taken care of at the sleep clinic, while the workshop participants were to a greater degree left to manage themselves with minimal external support. For many participants, the initial awareness or suspicion of sleep apnoea arose not from their GP, but through suggestions from a spouse, partner, or another individual in their personal network, or media.

Sleep clinic interview participant, on the journey to get a referral:

*"It felt like I had to do most of the work myself; going to the GP, following up when nothing happened, and trying to figure out what to do next. Without my wife pushing me, I might not have done anything about it."*

The same participant expressed how this was contrasted once they arrived at the sleep clinic:

*"At [the sleep clinic] everything was connected. You didn’t have to go back and forth between departments. They handled everything, and it made the process so much easier."*

In the workshop, participants emphasised the importance of having the determination to take charge of your treatment, and persist even when the benefits are not immediately apparent:

*"But in my mind, I tell myself that I have the will to make it work. That’s the key for many. It’s uncomfortable for 14 days. But I have a fantastic life for 17–18 years afterward […]. Maybe you need to be told that there is an adjustment period. Yes, it’s uncomfortable. But you need to have some thresholds and create a development ladder for yourself."*

Those who experienced the CPAP treatment as ineffective expressed the need for alternative or more personalised solutions, often describing how they must advocate for themselves in meetings with healthcare personnel. This sentiment was especially prominent in the workshop group, where more participants experienced limited or no positive effects from the treatment. To illustrate, two workshop participants discussed their ongoing effort to get more help and information about co-morbidities that they thought could be a contributing factor their persistent symptoms:

*“And I don’t really get any follow-up at [the hospital]. That’s why I signed up for this, and maybe I’ll get some tips on what can be done. Because I think if they found something that helps, my blood pressure would go down.”*

One participant described his lengthy efforts to get treated for low metabolism that he believed could be contributing to his condition:

*“And then there’s an issue called metabolism, and the key term is low metabolism. I’ve known about it since 2012. There was an article about it. And since then, I’ve struggled with general practitioners to get proper treatment for low metabolism."*

### Healthcare providers as gatekeepers—an obstacle or an ally?

Insights from both the workshops and individual sleep clinic interviews highlight how interactions with healthcare personnel shape participants’ experiences. Feelings of being forsaken, as discussed in the previous theme, often stem from encounters with GPs in particular. The discussions highlight how GPs, in their role as gatekeepers and referrers to specialist services, can be perceived as either obstacles or allies. While some participants in both groups appreciated a supportive GP relationship, others reported feeling overlooked due to perceived gaps in knowledge or engagement, aspects that they felt could possibly contribute to delays in further diagnosis and treatment.

Workshop participant:

*“My experience was first with my general practitioner. He immediately said, "If you come here and tell me that you snore, we have to take it seriously." That’s almost exactly what he said. And he pointed out that snoring itself wasn’t the problem—it was if there were apnoeas involved. But to find that out, I had to be evaluated. I felt very well received, and most importantly, I felt taken seriously, and things moved along quite quickly.”*

Participants in the sleep clinic interviews highlight positive interactions with the sleep clinic staff, citing clear instructions and responsiveness, and one-to-one consultations with both nurses and doctors. The sleep clinic is often seen as an "ally" in navigating care:

*"It felt like they were really looking out for me—making sure the machine worked, adjusting the settings, and answering any questions I had. It was a very personal approach."*

The workshop participants were more likely to describe GPs and other HCPs as obstacles, with limited guidance on managing the condition or accessing treatment.

Workshop participant:

*"[…] I don’t have a general practitioner who cares at all."*

One workshop participant emphasised the need to find a suitable GP;

*"You have to find one that works, someone you feel you have a good dialogue with, and who understands what’s wrong with you—what you experience as wrong, at least. So, there is a user responsibility in this, I think. If I had been dissatisfied with my general practitioner, I would have changed fairly quickly”*

Many participants in the workshops highlight the absence of structured follow-up and suggest regular check-ins to improve care and treatment adherence. In contrast, the participants in the sleep clinic interviews describe the regular and responsive follow-up care they received in the first year as positive, and empowering for handling challenges after the end of the scheduled follow-ups, although some expressed worry about being left to their own devices.

Workshop participant:

“​​​​*I miss having periodic CPAP cheques. If you've been given one, it should be followed up by the healthcare system.*”

Sleep clinic interview participants, after the final 1-year follow-up appointment:

“*If something goes wrong now, I’m not sure where to turn quickly. Waiting for my GP or the help centre feels like a long process.”*

“*I wish there was a routine follow-up schedule after the first year. It would help catch problems early and make me feel less on my own.”*

Among the sleep clinic interview participants, the consensus on the use of remote monitoring (AirView™) is positive, offering convenience, proactive care and reassurance:

“​​​​​*I think the [remote] monitoring made me stick to using the CPAP. I knew they could see if I wasn’t using it properly, and that accountability was helpful.”*

Some participants also offered suggestions for how the data could be used to optimise the follow-up:

“*It’s great they can see the data, but it would be even better if I had more regular, scheduled check-ins based on that data.”*

### Life on hold—the emotional and social impact

The emotional and social impact of sleep apnoea on quality of life is evident throughout the conversations in both groups. Several participants described the treatment as life-changing, saying they felt as though they had been given a new life and could no longer imagine living without the CPAP machine. These experienced benefits appeared to help them cope with some of the challenges the device can pose to social life and relationships. In contrast, those who continued to experience persistent daytime symptoms and saw little or no benefit from treatment expressed deep frustration and disappointment.

For many of the participants in both groups, efficacious CPAP treatment offers a sense of reclaiming life, restoring sleep quality and improving relationships after years of struggling with severe daytime symptoms, some of which had led to long-term sick leave. Many expressed how the treatment enabled a return to daily life and functioning, not only for themselves, but also their spouses and other family members, often referring to “we” or “us”:

Workshop participants:

“​​​​​​*For us, it’s been… I have my wife’s support. She’s also gotten a much better life after I got the mask.”*

Another participant expressed:

*“The machine is not exactly sexy, but wow, what a great life we’ve had since getting it.”*

However, for others, it introduces social stigma, emotional and social challenges, including impacts on family members:

*“What I think about in relation to relatives is that they should be aware of the general consequences of sleep apnoea. And also, everything surrounding the treatment, like having to carry equipment around. Additionally, when the treatment isn’t 100% successful, you still struggle with side effects. And those side effects also affect your relatives.”*

Some of the participants in the workshop group discussed how high hopes for treatment success can lead to feelings of disappointment when outcomes fall short, affecting emotional well-being. This experience seemed to be exacerbated by the very transformative experiences some of the other participants described. Participants describe having their expectations tied to similar testimonies of improved quality of life following sleep apnoea treatment.

There was a clear desire to express that one-size does not fit all, and that the current focus on CPAP efficacy lack nuance:

*“I would have told everyone if it had worked for me, that I got a new life. I would have taken every opportunity to tell people. But that’s not the whole picture.”*

## Discussion

The themes identified in our research align with much of what is reported in the existing literature. A meta-synthesis of qualitative studies on experiences with CPAP treatment for sleep apnoea found that patients often faced delays in receiving referrals from GPs, largely due to limited public and professional awareness (Brown et al., 2021). The synthesis also highlighted common challenges related to discomfort and the difficulty of adjusting to and maintaining use of the machine. At the same time, it emphasised the need for perseverance to benefit from treatment, as well as the resulting improvements in quality of life, often leading to a sense of dependency on the CPAP machine.

A notable observation is the contrast between experiences in more integrated settings and those in fragmented pathways, which may indicate opportunities for more coordinated, patient-centred approaches, an area we will explore further in the Co-Design phase. Our results revealed that the participants who underwent assessment, diagnosis and follow-up at the sleep clinic had a more positive experience of the journey as well as the outcome, in some instances contrasted by their experience from previous attempts at other institutions. While both groups share common struggles with CPAP use, maintenance, and navigating healthcare, participants in the individual interviews may have benefitted from a more cohesive system with faster diagnosis, more tailored and personalised care including one-to-one meetings with doctors and nurses, and proactive support. The workshop participants often faced more fragmented care, delayed treatment, and limited follow-up, which may have negatively impacted their experiences and outcomes. Some workshop participants expressed profound feelings of despair over not having reached a resolution with their diagnosis. This emotional burden highlights the need to better understand which aspects of the care journey can be improved to support those who face difficulties with adjustment and adherence, particularly patients dealing with additional or co-morbid conditions that may limit their ability to benefit fully from treatment. These experiences point to the need for more personalised and flexible approaches to care. The broader literature underscores the need for more personalised, patient-centred care, highlighting the vital role of clinicians, interdisciplinary management, and closer follow-up in effectively addressing this prevalent condition and potential comorbidities (Brown et al., 2021; Chua et al., 2023). The perception of remote monitoring (AirView™) among participants from at the sleep clinic suggests that digital service models can be part in facilitating more personalised care by providing convenience, reassurance, and a sense of accountability. Participants also identified opportunities for more proactive use, such as triggering regular, tailored check-ins based on the data. These insights highlight potential for remote monitoring to enhance follow-up, supporting improved treatment adherence.

Another point of reflection from our analysis is participants´ dependency on the CPAP machine. Although technology enabled home-based care is often associated with empowerment, autonomy and security (Leonardsen et al., 2020), participants described a dependence on the CPAP machine, which, while giving them symptom relief, also introduces a burden related to maintenance as well as a degree of vulnerability and anxiety related to potential device malfunction or unavailability. This tension is further illustrated by how the two groups attribute overcoming issues during the adaption phase differently; the workshop participants to their own persistence, while the participants in the sleep clinic interviews to the closer and more proactive support they received at the sleep clinic. Experiences with dependence on home-based care technologies has been explored in literature that highlight the experiences of patients with COPD and those who are in need of dialysis. These studies explain the ranging and often contrasting experiences patients have with being dependent on a technology-based therapy as both a lifeline and a toll, in how it upholds but also imposes on daily life and functioning, both practically and mentally (Elisabeth Arnold et al., 2011; Jacquet & Trinh, 2019; Martin-McDonald, 2003). Exploring how patients navigate dependency in the context of CPAP therapy could yield important insights to inform patient-centred approaches to sleep apnoea care.

The role of health care personnel, and the role of GPs in particular, in managing OSA as highlighted in this study has been investigated elsewhere (Bartlett et al., 2008; Devaraj, 2020; Hoon et al., 2021; Martins et al., 2019; Schotland & Jeffe, 2003). Recent studies assessing the knowledge, skills, attitudes and practices among primary care physicians and specialists involved in managing OSA in other parts of the world find that despite progress over the past decades, there is a need to improve interdisciplinary cooperation to detect and treat patients with OSA more effectively. The studies show that although doctors have adequate knowledge, they lack the confidence to manage OSA (Devaraj, 2020; Martins et al., 2019). The experiences of the participants in our study reflect the need for GPs who are more confident in their role in coordinating care with specialist services in identifying, managing and following-up patients with OSA. Further research could usefully examine the experiences of GPs and other health care personnel in the Scandinavian context.

While the findings presented in this article will be explored further in joint workshops with healthcare personnel to co-design and propose a concrete service model, they already point to the benefits of more coordinated, proactive, and patient-centred approaches. The four themes identified, ranging from the duality of CPAP as lifeline and burden to the emotional and social impacts, will serve as generative insights in the next Co-Design phase. They will guide ideation by highlighting pain points (i.e., device maintenance, fragmented follow-up) and opportunities for personalisation (i.e., proactive check-ins, integrated remote monitoring), ensuring that proposed solutions directly reflect lived experiences uncovered in this study. Building on these insights, we suggest that a tentative implementation of a personalised service model for patients with sleep apnoea could include: faster GP referrals supported by professional education, formalised interdisciplinary collaboration between specialists and GPs (i.e., through shared care plans to ensure continuity in follow-up), individualised interventions with proactive, one-to-one consultations, integration of remote monitoring to tailor follow-ups, structured in-person and/or digital check-ins, personalised patient education on device use and maintenance, including contingency plans for device malfunction and managing dependence.

### Limitations

Our results may suffer from some limitations in sampling, participation and recruitment bias. The small and geographically limited sample may limit the generalisability of these findings. The participants who agreed to take part in the individual interviews may have been inclined to participate due to their positive experiences with the service. We only recruited those who showed up for their follow-up, missing the opportunity to learn from the experiences of those who did not show. The use of convenience sampling may have led the participants in the workshops to reflect both participation and recruitment biases; those who were recruited through the Norwegian Association of Sleep Disorders may reflect negative experiences and/or failed treatment, leading them to seek community and further help through the association.

The individual interviews for this study were conducted during the COVID-19 pandemic, which imposed some methodological limitations. Public health restrictions and recommendations prevented us from conducting face-to-face interviews and follow-up conversations with participants. As a result, all the interviews were carried out remotely, through video calls or telephone. While this allowed us to continue the research, the lack of in-person interaction may have limited the depth and richness of the data. Non-verbal cues, rapport-building, and opportunities for spontaneous elaboration were constrained. Additionally, the absence of face-to-face interaction may have influenced the emotional and social depth of participants’ responses, potentially limiting the expression of feelings and emotional nuance, or interpersonal dynamics that might have emerged. Furthermore, the inability to conduct follow-up interviews reduced our capacity to clarify or expand upon participants’ initial responses, which may have affected the overall depth and nuance of our findings. The methodological changes necessitated by the pandemic also delayed and extended the data collection period, which could have affected the consistency, depth, and accuracy of findings by altering contextual relevance or introducing memory or interpretive bias. To prevent this, the lead author conducted the interviews following the same interview guide and protocol for each interview, and no shifts in the approach were observed during the analysis.

Another limitation of this article is the misalignment between journal guidelines and Braun and Clarke’s recommendations for reporting reflexive thematic analysis. For example, Braun and Clarke recommend the use of “analysis” over “results”, as the latter is most often associated with outputs of statistical analysis (Braun & Clarke, 2024). This highlights a broader challenge for qualitative researchers seeking to maintain methodological integrity while conforming to publication guidelines. Additionally, we note that commonly applied quality criteria in qualitative health research, such as data saturation and resolution of discrepancies, are not aligned with the principles of Reflexive Thematic Analysis. In this article we have not sought data saturation, but depth, richness and range in data through multiple forms of engagement with stakeholders. Furthermore, discrepancies in the data are treated as an important analytical finding, which is reflected in the presentation of the results. Although we consider our analytic stance, which intentionally prioritises depth, variation, and the value of divergent perspectives, a strength, we acknowledge that it may differ from conventional journal expectations.

## Conclusion

This study highlights the contrasting experiences of people living with sleep apnoea. Their journey, including referral, assessment, diagnosis, CPAP-treatment, and follow-up is seen as vital and life-sustaining as well as a source of emotional and physical frustration, posing a burden on life and relationships. The participants expressed appreciation for the service offerings, clinical encounters, and the therapeutic benefits of the CPAP machine. However, they were also frustrated with the challenges it introduces, such as delays, discomfort, maintenance demands, and limited effectiveness, reflected in experiences of dependency, abandonment, and disappointment. Positive experiences were associated with access to more personalised care and structured follow-up, while having to navigate the general healthcare settings independently was associated with feeling unsupported and alone in managing the condition.

Our analysis highlights the need for a service model more tailored and responsive to the diverse needs and broader physical and psychosocial realities of people living with obstructive sleep apnoea. These exploratory findings point to opportunities for more personalised and coordinated care and will inform subsequent co-design work with stakeholders to develop concrete suggestions for improved service models.

## Data Availability

The dataset generated and analysed during the current study is in Norwegian, and access is restricted to protect the privacy of participants. Anonymized data may be shared upon reasonable request to the corresponding author, subject to confidentiality requirements.

## Appendix A

**Table A1.** Data analysis RTA.

Data analysis following Braun and Clarks Framework for RTA (2019)			
Step	Activity/Description	Participant validation	Research team validation
**1. Familiarisation with the data**	The first author listened to audio recordings and read transcripts from workshops and interviews to become familiar with the data. Initial observations were noted, reflecting key research questions such as experiences with current service offerings, patient concerns, challenges, and desired improvements.	Not involved at this stage.	Initial observations were documented and discussed with the research team to support reflexivity.
**2. Generating initial codes**	Codes were generated inductively from smaller sets of data that shared the same meaning, staying close to the language of participants (semantic coding). For example, repeated words or phrases such as “new life” were coded.	Not directly involved.	Codes, transcripts, and early interpretations were reviewed and discussed in regular research team meetings to ensure analytical rigour.
**3. Searching for themes**	Candidate themes were formed based on the initial codes. Analysis incorporated a deductive element as data were considered through the lens of the research questions and service design, focusing on experiences with the service rather than personal factors.	Preliminary themes from workshop 1 were presented to participants in workshop 2 to review, validate, and provide feedback.	Multiple research team members reviewed the coding structure and emerging themes to enhance reflexivity and strengthen interpretation.
**4. Reviewing themes**	Themes were revised based on participant feedback from workshop 2. Some codes became themes themselves, while others merged into “new” themes. Additional data emerging during interviews were incorporated into the dataset for further analysis.	Participants actively engaged in validating and refining themes during workshop 2, ensuring they stayed close to their language and experiences.	Research team iteratively reviewed revisions to ensure coherence and alignment with the data.
**5. Defining and naming themes**	Themes were defined and named to reflect overarching meanings. Analysis of workshop and interview data was integrated to generate final themes. Discrepancies between datasets were interpreted as meaningful differences informing the results.	Participant validation occurred in workshop 2; due to the pandemic, interviews were not reviewed with participants.	Research team confirmed that the themes were sufficiently developed, coherent, and represented meaningful interpretations of the data.
**6. Producing the report**	The final themes were presented as generative insights to inform future co-design workshops and tangible service improvements.	Participants will further explore and develop these insights in forthcoming workshops to ensure outputs reflect patient experiences and clinical feasibility.	Research team authored the report, integrating insights across workshops and interviews, ensuring rigour and reflexivity.
